# Graphical analysis of pH-dependent properties of proteins predicted using PROPKA

**DOI:** 10.1186/1472-6807-11-6

**Published:** 2011-01-26

**Authors:** Michał Rostkowski, Mats HM Olsson, Chresten R Søndergaard, Jan H Jensen

**Affiliations:** 1 Department of Chemistry, University of Copenhagen, Universitetsparken 5, 2100 Copenhagen, Denmark

## Abstract

**Background:**

Charge states of ionizable residues in proteins determine their pH-dependent properties through their pK_a _values. Thus, various theoretical methods to determine ionization constants of residues in biological systems have been developed. One of the more widely used approaches for predicting pK_a _values in proteins is the PROPKA program, which provides convenient structural rationalization of the predicted pK_a _values without any additional calculations.

**Results:**

The PROPKA Graphical User Interface (GUI) is a new tool for studying the pH-dependent properties of proteins such as charge and stabilization energy. It facilitates a quantitative analysis of pK_a _values of ionizable residues together with their structural determinants by providing a direct link between the pK_a _data, predicted by the PROPKA calculations, and the structure via the Visual Molecular Dynamics (VMD) program. The GUI also calculates contributions to the pH-dependent unfolding free energy at a given pH for each ionizable group in the protein. Moreover, the PROPKA-computed pK_a _values or energy contributions of the ionizable residues in question can be displayed interactively. The PROPKA GUI can also be used for comparing pH-dependent properties of more than one structure at the same time.

**Conclusions:**

The GUI considerably extends the analysis and validation possibilities of the PROPKA approach. The PROPKA GUI can conveniently be used to investigate ionizable groups, and their interactions, of residues with significantly perturbed pK_a _values or residues that contribute to the stabilization energy the most. Charge-dependent properties can be studied either for a single protein or simultaneously with other homologous structures, which makes it a helpful tool, for instance, in protein design studies or structure-based function predictions. The GUI is implemented as a Tcl/Tk plug-in for VMD, and can be obtained online at http://propka.ki.ku.dk/~luca/wiki/index.php/GUI_Web.

## Background

The pH dependence of important protein properties such as binding affinity, catalytic activity, solubility, charge and stability is determined by ionizable residues [1-3]. Thus, it is of great importance for researches to have access to a reliable description of these residues. Protonation states of ionizable groups can be described with titration curves and ionization constants (pK_a _values). Because pK_a _values are difficult to obtain experimentally, especially for large biological systems, several software packages have been developed to predict them based on the protein structure [4-6]. PROPKA [7-9] is one of the popular protein pK_a _prediction software packages mainly because of its speed and accuracy compared to other methods [4,6], but also because it offers a structural rationalization of the predicted pK_a _values.

PROPKA computes the pK_a _values of the ionizable residues in a protein by determining a perturbation to the model pK_a _value, pK_model_, due to the protein environment [7-9]:

(1) $${\text{pK}}_{\text{a}}={\text{ pK}}_{\text{model}}+\Delta {\text{pK}}_{\text{DS}}+\Delta {\text{pK}}_{\text{HB}}+\Delta {\text{pK}}_{\text{CC}}$$

This perturbation comes from the desolvation penalty (DS), back-bone and side-chain hydrogen bonds (HB), and interactions with other charged groups (CC). The functional form of these terms and the associated parameters are determined empirically, and the relationship between the perturbation and the structure is described by simple distance and angle dependent functions in order to be evaluated with minimal computational effort, and to make analysis tractable also for large proteins or protein complexes. Results of the PROPKA calculations are saved in a formatted text file containing the pK_a _and pK_model _values for each ionizable residue as well as corresponding lists of all interactions contributing to the pK_a _shifts (equation 1). The PROPKA output file also contains the total charge of the protein and the pH-dependent free energy of unfolding, both as functions of pH. The latter can be obtained from the difference in the total protein charge between the folded and unfolded state at a given pH [10,11]:

(2) $$\Delta {\text{G}}_{\text{U}}\left(\text{pH}\right)=\Delta {\text{G}}_{\text{U}}\left({\text{pH}}_{\text{ref}}\right)+\text{1}.\text{36}\int_{{\text{pH}}_{\text{ref}}}^{\text{pH}}\left({\text{Q}}_{\text{U}}-{\text{Q}}_{\text{F}}\right)\text{dpH}$$

Here, ΔG_U_(pH_ref_) is the unfolding free energy at a reference pH, and the latter term is the pH-dependent change in the unfolding free energy related to the change in protein charge Q between two folding states. Thus, the perturbed protein pK_a _values are used to calculate the charge of the folded protein, whereas pK_model _values are used for the unfolded state.

The results from the PROPKA calculations can be very helpful, and give detailed information about the influence of the protein environment on the ionizable groups. Nevertheless, the PROPKA output does not provide a direct link between obtained pK_a _values and the three-dimensional structure of the studied system. In order to complete analysis of the ionizable residues one needs to make a separate search of these residues together with the interactions determining their pK_a _values by hand, using software for visualizing biomolecules. Furthermore, studying raw text data for larger sets of structures can easily become a difficult, complex and time-consuming task.

The PROPKA Graphical User Interface (GUI) presented in this paper is developed to facilitate exploration of the pH-dependent protein properties in a convenient manner by providing a direct link between the structure and the pK_a _data, predicted by the PROPKA calculations, via the Visual Molecular Dynamics (VMD) program [12]. Our interface is an easy-to-use tool to identify and rationalize residues with unusual pK_a _values or those significantly contributing to the free energy of unfolding. The PROPKA GUI is designed to facilitate the use of the PROPKA program and interpreting its results both for the user's convenience and to increase accessibility to the PROPKA approach for a wide range of researchers. Additionally, our GUI allows for comparative studies of the pH-dependent properties of many structures together, which can be used to rationalize the differences in these properties between homologous structures.

## Implementation

The PROPKA GUI is written in the Tool Command programming Language with the Tk graphical user interface (Tcl/Tk) as a platform-independent plug-in for the VMD program. The VMD program was chosen as a host application for the PROPKA GUI as it offers a great versatility of options and tools for analyzing biological structures, and also because it provides the Tcl/Tk environment as an extension of the VMD core code functionality without the need of making any additional installations. Besides, Tcl/Tk gives a wide range of users an easy but powerful tool to develop their own programs or scripts, or to extend already existing ones. The PROPKA GUI requires the VMD package to be installed on the user's computer. VMD can be obtained online at http://www.ks.uiuc.edu/Research/vmd/. The current version of our GUI is available as a single file that has to be copied into the VMD plug-ins directory and adding only one line into the VMD starting script makes the PROPKA GUI available from the menu in the main VMD window. The PROPKA GUI source-code, which is freely distributed under the GNU General Public License (GPL), installation instructions, documentation, and a screencast tutorial are available on the web at http://propka.ki.ku.dk/~luca/wiki/index.php/GUI_Web.

**Figure 1:**
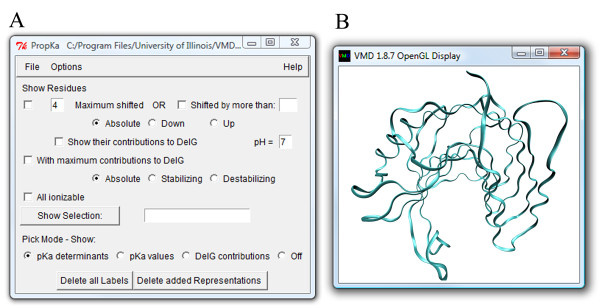
**The PROPKA GUI**. (A) The main window of the PROPKA GUI for accessing general pK_a _information about the ionizable residues and their contributions to the pH-dependent free energy of unfolding. (B) The structure display window of VMD for an interactive access to the pK_a _data for particular ionizable residues. It depicts the structure of *Bacillus circulans *xylanase [PDB:1XNB] as it is displayed, by default, after the structure and the pK_a _data are loaded, using simplified-style drawing method.

The GUI extracts and visualizes data from the PROPKA output file. The pK_a _calculations can be performed online at http://propka.ki.ku.dk/, or locally, via the GUI, if the PROPKA program is installed on the same computer. By default, the pK_a _data from the PROPKA output file and corresponding structure, contained in a separate Protein Data Bank (PDB) [13] file, are loaded simultaneously. pK_a _values and their determinants are assigned to the appropriate residues and can be accessed interactively either through the main PROPKA GUI window (Figure 1A) or through the structure display window of VMD (Figure 1B). It is also important to note that the data from the PROPKA output file is assigned to residues of the current top molecule in VMD, which allows for loading pK_a _data for all proteins in VMD separately. This provides the user with an access to the pK_a _data for many proteins within the same instance of VMD. Going further, such accessibility to the pK_a _information together with the VMD MultiSeq tool [14], which allows for structural alignment of homologous proteins, makes the PROPKA GUI a convenient tool to rationalize the differences in the pH-dependent properties between structurally-related proteins.

All residues and graphical objects displayed using the PROPKA GUI such as ionizable residues, pK_a _determinants, ligands, etc., depending on their type, are shown using pre-defined sets of VMD settings and representations. These representations can easily be accessed and modified in the "Graphical Representations" window of VMD. In order to make the PROPKA GUI more convenient to use, the user can also easily display the desired VMD selections, or remove previously shown, directly from the GUI. By default, all labels displaying the desired pK_a _information in the structure display window of VMD are drawn using different sets of colors for each molecule. Moreover, corresponding labels for different loaded structures, depending on their molecule ID in VMD, are shifted relative to each other to increase their readability in the case of overlapping residues, which considerably facilitates using the GUI for comparative protein studies. Additionally, the information shown in the structure display window is also printed in the VMD text console.

## Results and Discussion

The PROPKA GUI compares the computed pK_a _values to pK_model _values and can display residues with the largest pK_a _shifts. Based on equation 2, the GUI also computes and displays the contribution of each ionizable residue to the pH-dependent part of the free energy of unfolding. The GUI can therefore be used to display residues contributing the most to the unfolding energy. Moreover, it provides an interactive access to the pK_a _determinants listed in the PROPKA output file through the structure display window of VMD.

### Basic use

After installation of the PROPKA GUI plug-in, its main window (Figure 1A) can be accessed from the "Extensions" → "Analysis" menu in the main VMD window. By default, when the data from the PROPKA calculations and the appropriate PDB file are loaded, the structure is displayed automatically in the structure display window with a simplified-style drawing method (Figure 1B).

**Figure 2:**
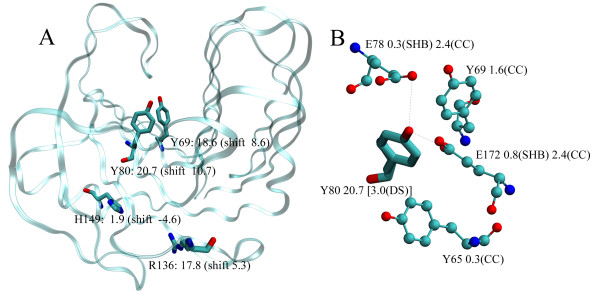
**Ionizable residues and pK_a _determinants in the xylanase structure**. (A) The figure shows four ionizable residues with the most shifted pK_a _values in the structure of *Bacillus circulans *xylanase [PDB:1XNB] displayed using the PROPKA GUI. Labels next to each residue give: one-letter residue code with its residue ID, the pK_a _value and the shift of the pK_a _from its model value. (B) When an ionizable residue is selected (in this case tyrosine 80), all of its pK_a _determinants are displayed together with their contributions to the pK_a _shift shown with the appropriate labels.

A user-defined number of residues with the most shifted pK_a _values, or with pK_a _shifts larger than a given threshold, can be displayed for the current top molecule in VMD simply by selecting the appropriate check box in the main GUI window. Figure 2A depicts the four residues with the largest pK_a _shifts in *Bacillus circulans *xylanase (BCX), [PDB:1XNB] [15], computed by PROPKA2. These residues are: tyrosine 80 and 69, arginine 136 and histidine 149 with pK_a _shifts of 10.7, 8.6, 5.3 and -4.6 pH units, respectively. This way, the user can easily visualize residues with the most perturbed pK_a _values, which can often facilitate identification of the key residues as, for example, in case of the active site residues [16,17]. In the same way, the residues contributing the most to the pH-dependent free energy of unfolding, at a given pH, or just the most stabilizing or destabilizing residues can be shown. It is also possible to display all ionizable residues in the protein at once or only the ones specified by the user. Moreover, the protein charge and the free energy of unfolding can be plotted as a function of pH through the "Options" menu, using the MultiPlot plug-in pre-installed in VMD.

More detailed pK_a _data can be accessed via the structure display window when the mouse picking mode is set to one of its "Label" actions. By default, when an ionizable residue or ionizable ligand atom is selected, all of its pK_a _determinants are displayed. In addition to the pK_a _value and the desolvation contribution for the selected residue, contributions to the pK_a _shift for all determinants are shown with the appropriate labels. Instead of displaying determinants, one can also choose to show only the pK_a _value or the contribution to the free energy of unfolding at a given pH. When the GUI interactive mode is disabled, VMD can be used in the standard way for analyzing the structure, making measurements of interatomic distances, angles, etc. As an example, we try to rationalize why the pK_a _value of tyrosine 80 in BCX is so extremely up-shifted compared to its model pK_a _value (20.7 compared to 10). By "clicking" on the residue with the mouse, we find that tyrosine 80 interacts strongly with three neighboring ionizable residues: glutamic acid 78, tyrosine 69, and second glutamic acid 172 (see Figure 2B). These contribute to raising the pK_a _value through unfavorable charge-charge interactions (CC) by 2.4, 1.6 and 2.4 pH units, respectively. Increase of the pK_a _value, but to a smaller extent, is also achieved by charge-charge interaction with tyrosine 65 and by hydrogen bonds to the side chains (SHB) of the mentioned glutamic acids. In addition, tyrosine 80 is buried in the protein, and therefore shielded from the solvent, which raises its pK_a _value by additional 3 pH units due to the desolvation energy (DS).

### Comparing structures

**Figure 3:**
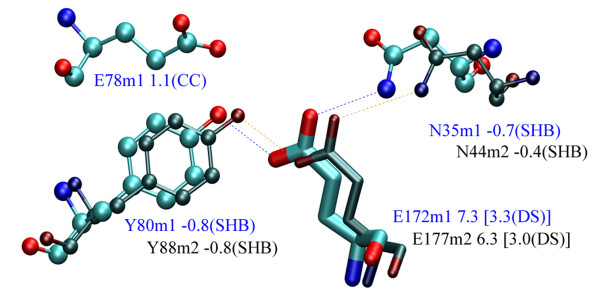
**Comparative study of active site residues**. By comparing pK_a _values of catalytic glutamic acids 172 and 177 together with their determinants, in two homologous xylanase structures [PDB:1XNB] and [PDB:1XYP], respectively, we find that the higher pK_a _of the former is mainly due to an extra charge-charge interaction with the glutamic acid 78.

Having all abovementioned options for accessing the pK_a _data in hand, the PROPKA GUI is also a useful tool for more complex and demanding analysis such as carrying out comparative studies of the pH-dependent properties for homologous proteins. After loading structures to compare together with the pK_a _information, and aligning their coordinates, using for example the MultiSeq tool from VMD, the differences in pK_a _values of particular residues can be rationalized simply by displaying these residues and their pK_a _determinants. An example of such comparison is shown in Figure 3 for the catalytic glutamic acids 172 (PROPKA2-computed pK_a _value of 7.3) and 177 (pK_a _= 6.3) for two xylanase structures, [PDB:1XNB] and [PDB:1XYP] [18], respectively. A cursory look on the pK_a _determinants of these two residues clearly shows that the difference results mainly from the additional, repulsive interaction with the charged group of the other catalytic nucleophile, glutamic acid 78, in the [PDB:1XNB] structure [11]. Such studies of homologous systems help to understand the key features underlying the differences in the protein properties. For example, they can help us to understand which residues and interactions are responsible for the extraordinary stability of extremophiles, or, for instance, which residues are crucial for certain reaction mechanism in enzyme-catalyzed reactions.

### Future development

Currently, the second version of the PROPKA GUI is under development. The main improvements will extend the basic GUI functionality by automated and user-friendly procedures for protein structure comparisons in order to better understand their pH-dependent properties. It will provide the user with a more advanced, but still convenient tool for a quick and robust analysis of structural differences determining different ionization constants of corresponding residues for large sets of homologous structures. Then, if needed, the tool can be used to suggest and verify desired modifications to the studied structures within seconds.

## Conclusions

Our newly developed PROPKA GUI is a powerful and convenient plug-in for VMD providing a direct link between the PROPKA-computed pK_a _values, their determinants and the three-dimensional structures. The GUI significantly improves ease of use of the PROPKA approach, and facilitates quick and easy investigation of the pH-dependent properties of proteins such as charge and stabilization energy as well as the separate pK_a _values and interactions determining them. It can easily be used to identify and rationalize ionizable residues with perturbed pK_a _values or contributing to the pH-dependent stabilization energy the most, either for a single protein or in comparison with other structures. This makes our GUI a helpful tool, for example, in the structure-based function prediction or protein design studies. Moreover, the PROPKA GUI is an open source code written in Tcl/Tk that can easily be customized whenever needed.

## Availability and requirements

• **Project name**: PROPKA GUI

• **Project home page**: http://propka.ki.ku.dk/~luca/wiki/index.php/GUI_Web

• **Operating system(s)**: Platform independent

• **Programming language**: Tcl/Tk

• **Other requirements**: VMD program installed

• **License**: GNU General Public License

• **Restrictions to use by non-academics**: None

## Authors' contributions

MR contributed to design, developing and testing software, and drafted the manuscript. MHMO and CRS contributed to design and software testing, provided support with the PROPKA program, and were involved in revising the manuscript. JHJ conceived the PROPKA GUI, contributed to design, and was involved in revising the manuscript. All authors read and approved the final manuscript.
